# A retrospective study on cervical intraepithelial lesions of low-grade and undetermined significance: evolution, associated factors and cytohistological correlation

**DOI:** 10.1590/1516-3180.2014.1322579

**Published:** 2014-04-01

**Authors:** Criseide Silva, Elia Cláudia Souza Almeida, Eliângela de Castro Côbo, Valéria Fátima Machado Zeferino, Eddie Fernando Cândido Murta, Renata Margarida Etchebehere

**Affiliations:** I MSc. Biomedic, Postgraduate Course on Pathology, Universidade Federal do Triângulo Mineiro (UFTM), Uberaba, Minas Gerais, Brazil; II MSc, PhD. Dentist, Discipline of Histology, Universidade Federal do Triângulo Mineiro (UFTM), Uberaba, Minas Gerais, Brazil; III MSc. Biomedic, Discipline of Special Pathology, Universidade Federal do Triângulo Mineiro (UFTM), Uberaba, Minas Gerais, Brazil; IV MSc. Pharmacist and Nursing Assistant, Universidade Federal do Triângulo Mineiro (UFTM), Uberaba, Minas Gerais, Brazil; V MD, PhD. Coordinator of the Postgraduate Course on Health Sciences, Universidade Federal do Triângulo Mineiro (UFTM), Uberaba, Minas Gerais, Brazil; VI MD, PhD. Surgical Pathology Service, Universidade Federal do Triângulo Mineiro (UFTM), Uberaba, Minas Gerais, Brazil

**Keywords:** Papillomaviridae, Risk factors, Uterine cervical neoplasms, Vaginal smears, Uterine neoplasms, Papillomaviridae, Fatores de risco, Neoplasias do colo do útero, Esfregaço vaginal, Neoplasia uterinas

## Abstract

**CONTEXT AND OBJECTIVES:**

Cervical cancer is an important cause of morbidity and mortality throughout the world. There is some controversy about the factors that may be associated with infection by the human papillomavirus (HPV) that may favor or protect against evolution from a low-grade intraepithelial lesion to a high-grade intraepithelial lesion or invasive neoplasia. The objective here was to evaluate the evolution of low-grade intraepithelial lesions and squamous or glandular lesions of undetermined significance, the associated factors and cytohistological correlations.

**DESIGN AND SETTING:**

Retrospective study conducted in a public tertiary-level university hospital.

**METHODS:**

Information was obtained by reviewing patient records and/or colposcopy reports. A statistical analysis was performed using logistic regression, calculating the odds ratio and applying chi-square tests.

**RESULTS:**

Of the 3390 patients, 409 evolved to high-grade intraepithelial lesions, of which 354 had an initial diagnosis of HPV infection, 27 of squamous atypia of undetermined significance, 22 of low-grade intraepithelial lesions with or without cytological diagnosis of infection by associated HPV and six of glandular cell atypia of undetermined significance.

**CONCLUSIONS:**

*Lactobacillus sp* and bacterial vaginosis on the smears, smoking and immunodepression were factors associated with evolution. A single partner, use of hormonal contraceptives, lower parity, age and a cytological diagnosis of cytolytic vaginosis, *T. vaginalis, Candida sp *or cocci were factors associated with protection. With regard to cytohistological correlation, there was a 74.08% agreement among patients with high-grade lesions and a biopsy obtained during the same period.

## Abstract (pt)

**CONTEXTO E OBJETIVOS:**

O câncer do colo uterino é importante causa de morbidade e mortalidade no mundo. Existem controvérsias sobre fatores que podem estar associados à infecção por papilomavírus humano (HPV) e favorecer ou proteger contra a evolução de lesão intraepitelial de baixo grau até lesão intraepitelial de alto grau ou neoplasia invasiva. O objetivo aqui foi de avaliar a evolução de lesões intraepiteliais de baixo grau e de significado indeterminado, escamosas ou glandulares, os fatores associados e as correlações citohistológicas.

**TIPO DE ESTUDO E LOCAL:**

Estudo retrospectivo realizado em hospital universitário público terciário.

**MÉTODOS:**

As informações foram obtidas por meio de revisão de prontuários e/ou fichas de colposcopia. A análise estatística foi realizada por meio de regressão logística, calculando-se o *odds ratio* e aplicando-se o teste qui-quadrado.

**RESULTADOS:**

Das 3.390 pacientes, 409 evoluíram para lesão intraepitelial de alto grau, das quais 354 tinham diagnóstico inicial de infecção por HPV, 27 de atipias escamosas de significado indeterminado, 22 de lesão intraepitelial de baixo grau com ou sem diagnóstico citológico de infecção por HPV associado e 6 de atipias em células glandulares de significado indeterminado.

**CONCLUSÕES:**

*Lactobacillus sp* e vaginose bacteriana nos esfregaços, tabagismo e imunodepressão foram fatores associados com a evolução. Único parceiro, uso de contraceptivo hormonal, baixa paridade, idade e diagnóstico citológico de vaginose citolítica, *T. vaginalis, Candida sp* ou cocos foram fatores associados a proteção. Com relação à correlação citohistológica, encontramos concordância de 74,08% nas pacientes com lesão de alto grau e biópsia no mesmo período.

## INTRODUCTION

The human papillomavirus (HPV) plays a central role in cervical carcinogenesis, and around it revolve various factors that directly or indirectly influence whether or not changes in the cervical squamous epithelium occur that can evolve into cancer.^1^ Among the factors most studied are immunological factors, smoking, age, pregnancy, use of hormonal contraceptives, color and microbiota. All these, to a greater or lesser extent, appear to boost the virus's action in host cells and to facilitate carcinogenesis. The high rate of spontaneous regression in cases of HPV infection and the small percentage that evolve into invasive neoplasia suggest that viral infection by itself is not sufficient and that other variables are involved in this process.^1^

Information about the prevalence of HPV is most frequently obtained at the start of an individual's sex life, i.e. during adolescence or at around 20 years of age.^2^ This infection is fleeting in most cases, without clinical manifestation and can become spontaneously cured. There is very large variability in the incidence of infection among white and black women within the same population, although this pattern seems to have become more frequent over recent years.^3^

In the vagina's normal microbiota, *Lactobacillus sp *produces acidic pH (3.8 to 4.5), which inhibits the growth of various other kinds of bacteria. On the other hand, vaginal content in which there is an absence or low concentration of *Lactobacillus sp *may be associated with pathogenic processes.^4^ Therefore, this bacterium has an important role in infection control, in cytolytic vaginosis and in maintenance of a healthy genital tract. 

Smoking can also be correlated with higher incidence and persistence of HPV infection and evolution to dysplasia/carcinoma *in situ* and invasive neoplasia.^5^ Its importance in oncogenesis is already well known, and high concentrations of tobacco derivatives such as nicotine have been observed in the cervical area.^6^ ^-^ ^8^ Women with multiple sexual partners, who start sex activity early and who are smokers or the partners of smokers also present higher risk of developing cervical intraepithelial neoplasias.^3^

The greater frequency of HPV infection among pregnant women than among non-pregnant women suggests that pregnancy is a risk factor for this infection. The maximum clinical expression of infection occurs during gestation, with rapid regression during the puerperium. This increase in incidence may be explained by immunological modulation or by the influence of hormonal factors during gestation. It is known that gestation gives rise to imbalance in the vaginal microbiota, thus favoring infections such as HPV, as well as other infectious agents.^9^

## OBJECTIVES

To analyze the evolution to high-grade intraepithelial lesion, related risk or protection factors and cytohistological correlations among patients with cytological diagnoses of HPV infection, low-grade intraepithelial lesion with or without an association with HPV, squamous cell atypia of undetermined significance or glandular cell atypia of undetermined significance.

## METHODS

This study was approved by the university's Ethics Committee on March 28, 2008 (protocol number 1032). Among all the patients followed up within Gynecology and Obstetrics Department at a public tertiary-level university hospital between 1995 and 2000, we reviewed the records and/or colposcopy reports of 3,390 patients with diagnosis of low-grade intraepithelial lesions or of lesions of undetermined significance: 1,398 (41.24%) had a cytological diagnosis of HPV infection; 73 (2.15%) had low-grade intraepithelial lesions with or without an associated diagnosis of HPV infection; 1,689 (49.83%) had squamous cell atypia; and 230 (6.78%) had glandular cell atypia of undetermined significance. We sought information about age, color, age when sexual activity started, number of partners, number of pregnancies, smoking, use of hormonal contraceptives, immunosuppression, other infections or associated changes (cytolytic vaginosis, *Trichomonas vaginalis*, bacterial vaginosis, *Candida* sp and cocci), by reviewing the records and/or colposcopy reports.

We performed a statistical analysis using logistic regression, odds ratio calculations and chi-square tests, seeking to evaluate whether these factors were or were not associated with a risk of or protection against evolution of the lesions. Of the 409 patients who evolved cytologically to high-grade intraepithelial lesions, only 297 (72.62%) had undergone a biopsy at our service during the same period.

## RESULTS

Among the 409 patients who evolved to high-grade intraepithelial lesions, 354 (86.55%) had an initial diagnosis of HPV infection, 22 (5.38%) had low-grade intraepithelial lesions with or without a diagnosis of HPV infection, 27 (6.60%) had squamous cell atypias of undetermined significance and 6 (1.47%) had glandular cell atypias of undetermined significance. The average age of the patients with a cytological diagnosis of HPV infection was 28.52 years (± 11.04); the average age of those with a diagnosis of HPV infection that evolved into a high-grade intraepithelial lesion was 33.80 years (± 12.95); and the average age of those who evolved and did not have a diagnosis of HPV infection was 30.23 years (± 11.30).

Among the 1,432 patients (42.24%) with a diagnosis of HPV infection or low-grade intraepithelial lesions associated with a diagnosis of HPV infection, 336 (23.46%) presented evolution and 1,096 (76.54%) did not. Among the 1,958 patients (57.76%) who did not have a cytological diagnosis of HPV infection, 73 (3.73%) evolved and 1,885 (96.27%) did not. The difference in evolution between the two groups proved to be of extremely high statistical significance (P < 0.0001). 

When the risk factors evaluated were compared with whether or not the patient evolved to a high-grade intraepithelial lesion, we found that among the 509 smokers, 140 (27.50%) evolved and among the 107 immunodepressed individuals, 14 (13.08%) did. There was a cytological diagnosis of bacterial vaginosis for 711 patients, of whom 93 (13.08%) evolved to a high-grade intraepithelial lesion; 2,184 patients had a cytological diagnosis of *Lactobacillus sp*, of whom 214 (9.80%) evolved. The statistical analysis showed that smoking, immunosuppression, bacterial vaginosis and *Lactobacillus sp* were risk factors. Moreover, as expected, HPV infection was considered to be a risk factor for evolution. In evaluating the numbers of patients who used hormonal contraceptives (552; 84.40%), had a single partner (621; 86.01%), were infected with *Candida sp *(560; 89.03%), were infected with cocci (269; 89.67%), were infected with *Trichomonas vaginalis *(135; 88.82%) and presented cytolytic vaginosis (175; 92.11%), but who did not evolve to a high-grade intraepithelial lesion, we observed that these factors were associated with protection.

Parity and age were univariate analyses and were not included in the logistic regression. The statistical analysis showed that neither white skin nor pregnancy interfered with evolution. The factors studied and their associations with a risk of or protection against evolution to high-grade intraepithelial lesions are summarized in Table 1.

**Table 1.** Factors evaluated among patients with or without evolution from HPV, low-grade intraepithelial lesions or squamous or glandular lesions of undetermined significance to high-grade intraepithelial lesion, between 1995 and 200

Factor	Evolution n (%)	Without evolution n (%)	Total n (%)
Human papillomavirus	336 (23.46%)	1,096 (76.54%)	1,432 (42.24%)
Smoking	140 (27.50%)	369 (72.50%)	509 (15.01%)
Immunosuppression	14 (13.08%)	93 (86.92%)	107 (3.16%)
Bacterial vaginosis	93 (13.08%)	618 (86.92%)	711 (20.97%)
*Lactobacillus sp*	214 (9.80%)	1,970 (90.20%)	2,184 (64.42%)
Hormonal contraceptive	102 (15.60%)	552 (84.40%)	654 (19.29%)
Single partner	101 (13.99%)	621 (86.01%)	722 (21.30%)
*Candida sp*	69 (10.97%)	560 (89.03%)	629 (18.55%)
Cocci	31 (10.33%)	269 (89.67%)	300 (8.85%)
*Trichomonas vaginalis*	17 (11.18%)	135 (88.82%)	152 (4.48%)
Cytolytic vaginosis	15 (7.89%)	175 (92.11%)	190 (5.60%)
White color	267 (11.07%)	2,145 (88.93%)	2,412 (71.15%)
Pregnancy	70 (12.89%)	473 (87.11%)	543 (16.02%)

Only 297 of the 409 patients who evolved to high-grade intraepithelial lesions had undergone a biopsy performed within our service, with histological concordance of 74.07%. 

## DISCUSSION

The average age of the patients with diagnoses of HPV infection, low-grade intraepithelial lesion, atypias in squamous or glandular cells of undetermined significance, which evolved into highgrade intraepithelial lesions, was 33.80 years (± 12.95). Among the patients who did not evolve, the average age was 32.36 years (± 12.26). Evolution of the lesions is slow when it occurs, and the average time that elapses between infection and manifestation of a high-grade lesion or cancer is up to 15 years,10 thus explaining the higher average age of our patients who evolved.

Our data and that of similar studies^11^ ^-^ ^14^ confirm that HPV infection is fundamental to the development of high-grade intraepithelial lesions, thus making it a veritable precursor for cervical cancer. Even among the 73 patients (3.73%) who did not have a cytological diagnosis of HPV infection, we cannot completely discount this association, since we did not perform molecular biology tests, which are considered to be more sensitive and specific. Another study conducted in our region, also on patients with a cytological diagnosis of HPV infection, found a probability of evolution to high-grade intraepithelial lesions of 0.4%, over a four-year period. Nevertheless, the authors of that study were unable to determine the risk factors for persistence or evolution of the infection.^15^ Other authors have reported that around 10% of patients with the HPV infection present evolution.^13^ ^,^ ^16^ ^,^ ^17^

It is believed that the relationship between evolution to a high-grade epithelial lesion and presence of *Lactobacillus sp* stems from the high incidence of this microbiota, which is predominant in the vaginal environment,^18^ and not its actual interference in lesion evolution. In relation to smoking, 27.5% of the patients who reported this habit evolved, which was a statistically significant result. The importance of smoking to oncogenesis is already well known, and a high concentration of tobacco derivatives such as nicotine has been isolated in the cervical area. Smoking has been correlated with the incidence and persistence of HPV infection and its evolution to dysplasia, carcinoma *in situ *and invasive neoplasia.^6^

Cellular immune response seems to play an important role in curing HPV infection. There is a high prevalence of infection or pre-neoplastic lesions in people with compromised immune systems, such as those with renal transplants and individuals with acquired immunodeficiency syndrome.^19^ ^-^ ^21^ Another study conducted at our service demonstrated that there was strong expression of CD3+ lymphocytes in patients colonized by cervical intraepithelial neoplasia grade III (CIN III) who presented recurrence of the lesion, thus suggesting that these lymphocytes are of key importance in lesion evolution.^22^

We also observed that there was a positive association between bacterial vaginosis and the patients' evolution. In the literature, a significant association between HPV DNA and a microbiota indicative of bacterial vaginosis is shown. Some authors have suggested that bacterial vaginosis has an important role in the development of cervical neoplasia because of production of oncogenic nitrosamines from anaerobic bacteria, and also through stimulation of production of cytokines such as interleukin 1B. Another possibility that might favor evolution of lesions associated with bacterial vaginosis would be changes to vaginal pH, which our study was unable to evaluate, since it was retrospective and our service did not routinely perform evaluations on vaginal pH.^23^

With regard to other factors, hormonal contraceptives have been described in the literature as being associated with cell transformation and progression from low-grade to high-grade lesions.^24^ This was contrary to what our study observed, in which use of hormonal contraceptives was a protective factor. Having a single partner probably functions as a protective factor, as observed in our study. However, the behavioral patterns of these partners and their ages in relation to those of the women perhaps also need to be observed. Some authors have taken the view that these factors are as important as the number of partners.^25^ ^-^ ^27^

Infection with *Candida sp *also proved to be a factor protecting against evolution. Other authors have proposed that candidiasis might activate latent HPV infection.^28^ ^,^ ^29^ We believe that a very low pH, which favors infection by *Candida sp, *is one of the factors associated with this protection. A reduction in pH makes the vagina inhospitable to certain bacterial species^30^ and probably makes it difficult for HPV infection to evolve.

The presence of cocci in the vaginal microbiota also appears to confer protection against evolution. Their presence appears to be related mainly to inadequate hygiene habits and not to changes in vaginal pH.^31^ Utagawa et al. have suggested that socioeconomic status and inadequate hygiene are key factors for HPV infection.^32^ Despite this claim, we did not find any studies correlating the presence of cocci in cervicovaginal cytological samples with HPV infection and its evolution. 

Defining factors that can enhance or minimize cervical viral carcinogenesis is very important for clinical practice because these have an impact on development of the precursor lesions. However, further studies are needed to clarify the mechanisms of action of these factors.

## CONCLUSIONS

According to our study, we can conclude that Lactobacillus sp and bacterial vaginosis in smears, smoking and immunodepression were factors associated with evolution of low-grade intraepithelial lesions or lesions of undetermined significance to high-grade intraepithelial lesions. A single partner, use of hormonal contraceptives, lower parity, age and a cytological diagnosis of cytolytic vaginosis, T. vaginalis, Candida sp or cocci were factors associated with protection. With regard to cytohistological correlation, there was a 74.08% agreement among patients with high-grade lesions who had undergone a biopsy during the same period.
